# Growth differentiation factor‐15 as a prognostic biomarker in cancer patients

**DOI:** 10.1002/jcsm.12123

**Published:** 2016-05-22

**Authors:** Junichi Ishida, Masaaki Konishi, Masakazu Saitoh, Jochen Springer

**Affiliations:** ^1^ Innovative Clinical Trials, Department of Cardiology and PneumologyUniversity Medical Centre GöttingenGöttingenGermany

Growth differentiation factor‐15 (GDF15) is a member of the transforming growth factor beta superfamily and has been demonstrated to play multiple roles in various pathological conditions such as cancer, inflammatory diseases, cardiovascular diseases, and metabolic disorders.^1^ Furthermore, recent studies have shown that plasma levels of GDF15 increased in patients with chronic diseases described previously^1^ and that elevated GDF15 was associated with poor prognosis in patients with malignancy such as prostate cancer and upper gastrointestinal cancer.^2^, ^3^ In this study, Lerner *et al*. reported that elevated plasma GDF15 levels are associated with increased cancer‐related weight loss and decreased survival in cancer male patients.^4^

Cancer‐related weight loss has been shown to be an independent predictor for survival in cancer patients,^5^ which suggests that treatment for cancer cachexia might lead to the better outcome in cancer patients with weight loss. Recent studies have found several diagnostic biomarkers for loss of muscle mass such as N‐terminal peptide of procollagen type III, C‐terminal agrin fragment, leptin, ghrelin, and obestatin^6^, ^7^ and prognostic biomarkers in cancer patients such as matrix metalloproteinases, survivin, and butyrylcholinesterase.^8^, ^9^, ^10^ Although GDF15 might also be a useful prognostic marker in cancer patients, the small sample size, the heterogeneous population, and the selection bias could have some impacts on the findings in this study. To clarify the pathophysiological significance of GDF15 in cancer patients, validation studies on larger sample size including various cancer types and different pathological stages are needed. In addition, further research should demonstrate which biomarker including GDF15 and other novel biomarkers shows better diagnostic or prognostic performance or whether a multi‐marker approach is superior to each biomarker. Finally, it is of great research interest and clinical value whether these biomarkers could be the cancer therapeutic targets in this population.

## Acknowledgements

We acknowledge support by the German Research Foundation and the Open Access Publication Funds of the Göttingen University.

We certify that we comply with the ethical guidelines for publishing in the Journal of Cachexia, Sarcopenia and Muscle: update 2015.^11^
